# Sodium butyrate and sodium propionate inhibit breast cancer cell migration and invasion through regulation of epithelial-to-mesenchymal transition and suppression of MEK/ERK signaling pathway

**DOI:** 10.3389/fcell.2025.1535563

**Published:** 2025-03-12

**Authors:** Dania Mahmoud Kharazi, Louna Karam, Charbel El Boustany, José-Noel Ibrahim

**Affiliations:** ^1^ Department of Biological Sciences, School of Arts and Sciences, Lebanese American University (LAU), Beirut, Lebanon; ^2^ Department of Laboratory Science, Faculty of Public Health - Branch 2, Lebanese University, Fanar, Lebanon

**Keywords:** breast cancer, short chain fatty acids (SCFAs), migration, invasion, epithelial-to-mesenchymal transition (EMT)

## Abstract

**Objective:**

This study aims to investigate the roles played by NaB and NaP in breast carcinogenesis by elucidating their potential anti-metastatic effects in the context of tumor migration, invasion, and EMT regulation in two distinct breast cancer cell lines, MCF-7 and MDA-MB-231.

**Methods:**

The cytotoxic effect of both compounds on 3D spheroid formation was evaluated using a hanging drop assay. The anti-migratory and anti-invasive potentials of NaB and NaP were investigated through transwell migration and invasion assays. Moreover, their role in regulating epithelial-to-mesenchymal transition (EMT) was examined by assessing E-cadherin, vimentin, and β-catenin mRNA and protein expression levels through RT-qPCR and Western blot or flow cytometry. β-Catenin localization upon treatment was further visualized via immunofluorescence. Protein expression of MEK, p-MEK, ERK, and p-ERK was analyzed by Western blot.

**Results:**

Our results revealed a dose- and time-dependent impairment of spheroid formation in both cell lines, with NaB exerting a more potent effect than NaP. Both SCFAs were able to significantly inhibit migration and invasion of MDA-MB-231 cells following 24 h of treatment. Moreover, treatment with NaB or NaP altered the mRNA and protein profile of EMT-associated markers and abrogated the nuclear translocation of β-catenin. Finally, ERK and MEK phosphorylation was reduced in MDA-MB-231 and MCF-7 cells upon treatment with NaB, and less prominently with NaP.

**Conclusion:**

Our study highlights the promising therapeutic potential of NaB and NaP, providing insight into their inhibitory effects on 3D formation, migration, and invasion through EMT regulation and deactivation of MEK/ERK signaling in breast cancer.

## 1 Introduction

Breast cancer today is the most commonly diagnosed malignancy in women, accounting for 1 in 8 cancer diagnoses (Arnold et al., 2022). Despite considerable therapeutic success witnessed with conventional and targeted treatment options, breast cancer therapy remains challenged by tumor heterogeneity, drug resistance, and adversity of side effects, urging the need for more effective novel therapeutics. Treatment is also further complicated and rendered ineffective once the cancer metastasizes to distant body parts. A crucial hallmark for metastasis to occur is epithelial-to-mesenchymal transition (EMT) which entails the loss of epithelial characteristics such as cell polarity and cell-cell adhesion, and the gain of mesenchymal features, most notably migratory and invasive capacity (Kalluri and Weinberg, 2009). EMT contributes to breast cancer metastasis through various key regulators, notably the loss of E-cadherin, the gain of vimentin, and the translocation of β-catenin from cytoplasm to nucleus, where it promotes the constitutive transcription of EMT-inducing genes (Lai et al., 2020).

Among molecules being investigated in recent years, short-chain fatty acids (SCFAs), in particular sodium butyrate (NaB), have emerged as potential anti-cancer agents and garnered increasing research interest due to their inhibitory tumor effects in various cancers, particularly colon cancer (Jaye et al., 2022). SCFAs are small organic acids with less than six carbons produced by the gut microbiota during the anaerobic fermentation of partially and nondigestible polysaccharides (Al-Qadami et al., 2022).

NaB has been found to mediate its anti-cancer effect via signaling pathways that regulate cell proliferation, cell cycle, and apoptosis (Chen et al., 2019). Although research on NaB’s role in breast cancer is less comprehensive, few studies have demonstrated its ability to induce cell cycle arrest and apoptosis in different breast cancer models (Chopin et al., 2004; Wang et al., 2016; Salimi et al., 2017). Compared to NaB, research on the therapeutic potential of sodium propionate (NaP) as an anti-cancer agent is much more limited, particularly in breast cancer. However, recent work by Park et al. (2021) showed that NaP suppresses proliferation and induces apoptosis in MCF-7 cells, with significant suppression of tumor growth *in vivo* in mice bearing MCF-7 cell xenografts.

Interestingly, our research team has previously provided original evidence on the comparative roles of NaB and NaP in MCF-7 (Semaan et al., 2020) and MDA-MB-231 (Ibrahim et al., 2024) cell lines, which model the non-invasive luminal A subtype and the more aggressive triple-negative breast cancer subtype, respectively (Nohara et al., 1998). The findings demonstrated a time- and dose-dependent inhibition of cell proliferation by NaB and NaP, with NaB having a more potent effect than NaP and MDA-MB-231 more resistant to treatment than MCF-7. Interestingly, both compounds showed no significant toxicity or effects on the normal breast cell line MCF-10A, hence indicating their selectivity against cancer cells. Moreover, both SCFAs induced a G1 cell cycle arrest and apoptosis in MCF-7 and MDA-MB-231.

While such studies have elucidated the anti-cancer effects of these SCFAs on proliferation, apoptosis, and cell cycle regulation in breast cancer, their impact on metastasis has not been thoroughly investigated and the underlying mechanisms involved remain elusive. The current study builds upon our findings by delving into the anti-metastatic potential of NaB and NaP in breast cancer. Specifically, it aims to (1) investigate and compare the effect of NaB and NaP on the migration and invasion of MCF-7 and MDA-MB-231 cells, (2) evaluate their role in EMT regulation by examining the profile of E-cadherin, β-catenin, and vimentin, and (3) elucidate the potential molecular mechanisms involved.

## 2 Materials and methods

### 2.1 Cell culture

Breast cancer cell lines, MCF-7 and MDA-MB-231, were acquired from the American Type Culture Collection (ATCC). Cells were cultured as previously described (Semaan et al., 2020; Ibrahim et al., 2024), and they were counted and checked for viability, using the trypan blue exclusion method, prior to plating.

### 2.2 3D formation assay

To mimic *in vivo* tumor growth, cells were allowed to form 3D spheroids using the hanging drop method, which allows for the study of various tumor characteristics, including cell-cell cohesion and cell-substratum adhesion (Foty, 2011). MCF-7 (5 × 10^5^ cells/mL) and MDA-MB-231 (2.5 × 10^5^ cells/mL) cell suspensions were treated with increasing concentrations of NaB or NaP (1, 3, 5, 10, 15 mM). Four replicates of 20 μL droplets were pipetted on the inverted lid of a tissue culture dish, and 1X Phosphate Buffered Saline (PBS, Sigma-Aldrich) was added in the bottom to serve as a hydration chamber. Dishes were incubated at 37°C for up to 3 days to allow for spheroid formation. Spheroids were imaged using an inverted light microscope and measured using FIJI software (ImageJ).

### 2.3 Transwell assay

MCF-7 and MDA-MB-231 cell migration was analyzed using 24-well Transwell chambers (pore size: 8 µm, Corning). Cells (2.5 × 10^5^/mL) were loaded in the upper chamber containing serum-free medium, with or without NaB (2.5, 5, 10 mM) or NaP (5, 10, 15 mM). These concentrations were selected based on the IC50 values that we established in our previous studies after 72 h of treatment (NaB: IC50 = 1.26 mM for MCF-7 and 2.56 mM for MDA-MB-231; NaP: IC50 = 4.50 mM for MCF-7 and 6.49 mM for MDA-MB-231) (Semaan et al., 2020; Ibrahim et al., 2024). Notably, these concentrations were shown to be non-cytotoxic at 24 h, even at concentrations as high as 20 mM for NaB and 30 mM for NaP, ensuring that the observed effects were not confounded by cell death. The lower chamber contained DMEM media supplemented with 10% FBS. After 24 h, migrated cells were fixed with 4% paraformaldehyde (PFA) and stained with 0.5% crystal violet. For invasion assay, MDA-MB-231 cells were starved overnight in DMEM-0.5% FBS, plated in Matrigel-coated Transwell inserts, and incubated for 30 h. Invading cells were fixed with 4% PFA and stained with 4% crystal violet. Both migrated and invading cells were imaged using an inverted light microscope, and the absorbance of dissolved crystal violet in 1% SDS was measured at 570 nm.

### 2.4 Reverse-transcription quantitative real-time PCR (RT-qPCR)

MCF-7 and MDA-MB-231 (2 × 10^4^ cells/mL) were plated in 6-well plates, with or without NaB (2.5, 5, 10 mM) or NaP (5, 10, 15 mM). After 24 h, total RNA was extracted using the RNeasy Plus Mini kit (Qiagen). One µg of RNA was reverse transcribed using the iScript cDNA Synthesis Kit (Bio-Rad). q-PCR was performed using iTaq Universal SYBR Green Supermix (Bio-Rad) and primers (10 µM), selected *via* Primer-BLAST. Triplicate samples were analyzed, and relative expressions of E-cadherin, β-catenin, and vimentin were calculated by the 2^−ΔΔCt^ method, using *GAPDH* as reference. The primers (TIB Molbiol) used are as following: E-cadherin- F: 5ʹ-CCTCCTGAAAAGAGAGTGGA-3ʹ and R: 5ʹ-GTGTCCGGATTAATCTCCAG-3ʹ; β-Catenin- F: 5ʹ-AGGGATTTTCTCAGTCCTTC-3ʹ and R: 5ʹ-ACTGAACCTGACCGTACACATGCCCTCATCTAATGTCT-3ʹ; Vimentin- F: 5ʹ-GCTGCTAACTACCAAGACAC-3ʹ and R: 5ʹ-TCAGGTTCAGGGAGGAAAAG-3ʹ; and *GAPDH*- F: 5ʹ-AGCCTTCTCCATGGTGGTGAAGAC-3ʹ and R: 5ʹ-CGGAGTCAACGGATTTGGTCG-3.

### 2.5 Western blot

MCF-7 and MDA-MB-231 cells (1.25 × 10^4^ cells/mL) were plated in triplicates in 6-well plates, with or without NaB (2.5, 5, 10 mM) or NaP (5, 10, 15 mM) for 24 h. Total protein lysates were extracted using 1X RIPA (Merck) and 1X protease inhibitor cocktail (Sigma-Aldrich). Proteins were quantified using the DC Protein Assay (Bio-Rad), and 40 µg were separated by SDS-PAGE and transferred to PVDF membranes (Bio-Rad). Membranes were blocked with 5% non-fat milk in Tris buffered saline-Tween-20 (TBS-T, Bio-Rad), then incubated overnight at 4°C with the following primary antibodies (1:1000): E-Cadherin (CST, #14472), β-Catenin (CST, #8480), Vimentin (CST, #5741), ERK1/2 (CST, #9102), Phospho-ERK1/2 (CST, #9101), Phospho-MEK1/2 (CST, #9121), and MEK1/2 (CST, #9122). β-Actin (CST, #4970) was used as loading control. Blots were incubated with HRP-conjugated secondary antibodies, anti-mouse and anti-rabbit (1:2500, Promega) and visualized using the Clarity™ Western ECL Blotting Substrate (Bio-Rad) on ChemiDoc XRS+ (Bio-Rad). Bands were quantified using the Image Lab Software (Bio-Rad), and expression ratios were calculated relative to the loading control.

### 2.6 Flow cytometry

MDA-MB-231 cells (1.25 × 10^5^ cells/mL) were seeded in duplicates in 6-well plates, with or without NaB (2.5, 5, 10 mM) or NaP (5, 10, 15 mM) for 24 h. Following treatment, the cells were dissociated from the plates using collagenase V and vortexed to obtain a single-cell suspension. The cells were fixed in 2% PFA in 1X PBS for 10 min and then permeabilized using 0.1% Triton-X-100 in 1X PBS supplemented with 0.5% BSA for 15 min at 4°C. They were subsequently incubated with a monoclonal mouse E-cadherin antibody (CST, #14472) diluted 1:200 for 45 min. After two washes with 0.5% BSA, the cells were treated with a secondary goat anti-mouse IgG (H + L) antibody conjugated to Alexa Fluor 488 (Thermo Fisher Scientific, #2382186) at a dilution of 1:500 for 45 min. To evaluate non-specific binding, a mouse IgG1 isotype control, Alexa Fluor™ 488 (Thermo Fisher Scientific, #MG120) was employed. The stained cells were resuspended in 300 µL of PBS and analyzed using a BD Accuri™ C6 Flow Cytometer, recording a minimum of 10,000 events per sample. Data analysis was conducted using Accuri software.

### 2.7 Immunofluorescence

Cells were plated on glass coverslips in 6-well plates and treated with NaB (2.5 and 10 mM) or NaP (5 and 15 mM). After 24 h, cells were fixed with 4% PFA, permeabilized with 0.2% Triton-X-100 in PBS, and blocked in 0.1% Triton-X-100% and 10% FBS in PBS. Cells were then incubated with β-catenin (CST, #8480, 1:100), followed by Alexa Fluor 488 goat anti-rabbit secondary antibody (1:2,000). After washing, cells were stained with Hoechst 33,342 (1:1,000) and imaged using a Zeiss Observer Z1 widefield fluorescence microscope. The quantification of the nuclear-to-cytoplasmic ratio of fluorescence intensity was assessed using FIJI software (ImageJ). For each experimental condition, 10 randomly selected images were analyzed, resulting in the measurement of approximately 100 cells.

### 2.8 Statistical analyses

Statistical analyses were performed using GraphPad Prism 10 (GraphPad Software, Inc., San Diego, CA, United States). Comparisons between control and treatment groups were analyzed using One-Way ANOVA or Kruskal–Wallis followed by Dunn’s multiple comparison *post hoc* test. *P* values less than 0.05 were considered significant. All experiments were repeated at least three times.

## 3 Results

### 3.1 Treatment with NaB or NaP impairs 3D formation in a dose-dependent manner

**FIGURE 1:**
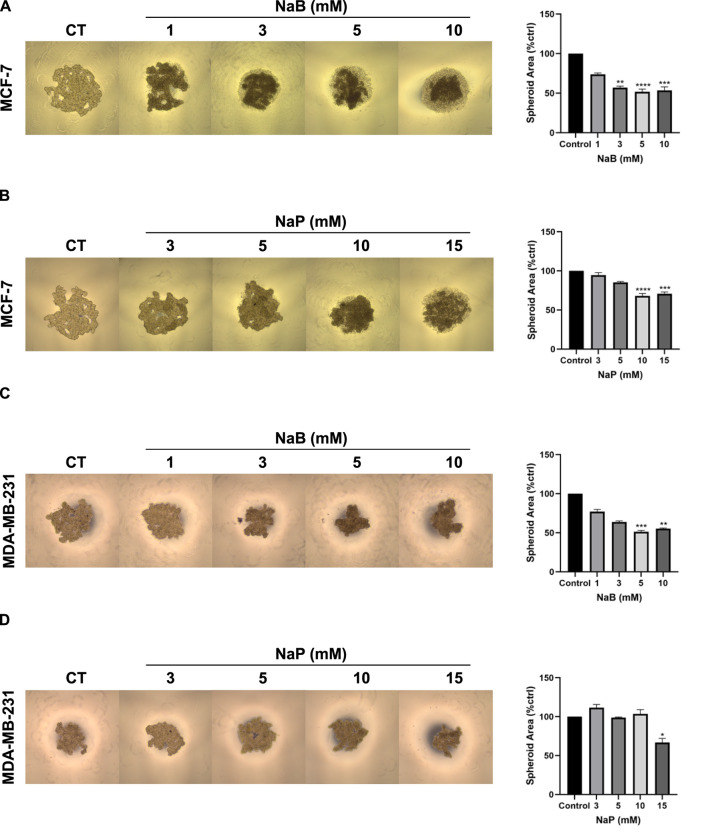
Effect of NaB and NaP on MCF-7 and MDA-MB-231 spheroid formation and growth. Representative images of MCF-7 **(A, B)** and MDA-MB-231 **(C, D)** spheroids imaged respectively at day 2 and day 3 after NaB and NaP treatment. Adjacent bar graphs (right panels) show spheroid area as percent control with respect to increasing concentrations of NaB (1, 3, 5, 10 mM) and NaP (3, 5, 10, 15 mM). Kruskal–Wallis test followed by Dunn’s multiple comparisons test were used to compare the different concentrations to the control. Values were represented as mean ± SEM from four independent experiments (n = 4), each with four replicates per condition. Significant differences were reported as * (p < 0.05), ** (p < 0.01), *** (p < 0.001), or **** (p < 0.0001).

MCF-7 spheroids formed within 48 h while MDA-MB-231 spheroids took 72 h. As shown in Figures 1A, B, MCF-7 spheroids treated with NaB or NaP were morphologically smaller in size and darker in color with progressively increasing SCFA concentrations compared to the control. Spheroid area was significantly reduced to 53.71% ± 4.36% with 10 mM NaB and to 70.84% ± 2.17% with 15 mM NaP, indicating a more potent effect of NaB than NaP. Similarly, MDA-MB-231 spheroids became smaller and darker with increasing concentrations of NaB (Figure 1C) and, to a lesser extent, NaP (Figure 1D). Significant reductions were noted at 5 mM (51.39% ± 1.67%) and 10 mM (55.27% ± 0.88%) NaB, and at 15 mM NaP (66.84% ± 1.67%).

### 3.2 Treatment with NaB or NaP reduces migratory and invasive capacity of MDA-MB-231 cells but not MCF-7 cells

**FIGURE 2:**
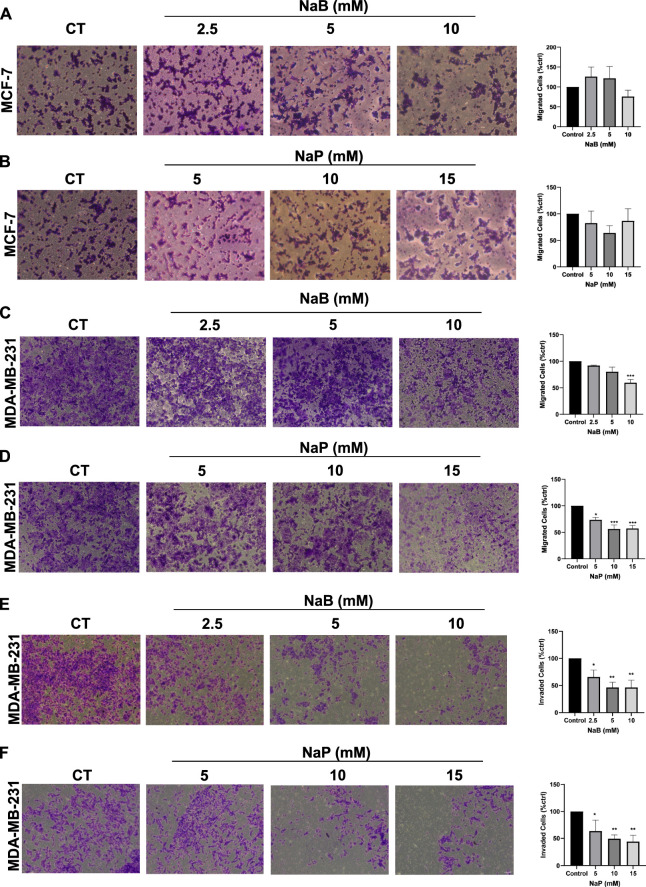
Effect of NaB and NaP on breast cancer cell migration and invasion using transwell assay. Representative images of migrated MCF-7 **(A, B)** and MDA-MB-231 **(C, D)** cells stained with crystal violet after 24 h of treatment with NaB (2.5, 5, 10 mM) or NaP (5, 10, 15 mM). Representative images of invading MDA-MB-231 cells **(E, F)** stained with crystal violet after 30 h of treatment with NaB (2.5, 5, 10 mM) or NaP (5, 10, 15 mM). Adjacent bar graphs (right panels) show the percentage of migrating or invading cells as compared to the control. Kruskal–Wallis test followed by Dunn’s multiple comparisons test were used to compare the different concentrations to the control. Values were represented as mean ± SEM from four independent migration experiments (n = 4) and three independent invasion experiments (n = 3). Significant differences were reported as * (p < 0.05), ** (p < 0.01), *** (p < 0.001), or **** (p < 0.0001).

The anti-migratory and anti-invasive effects of NaB and NaP on breast cancer cells were evaluated using non-cytotoxic concentrations, shown to not inhibit cellular proliferation at 24 h. Both SCFAs had no significant anti-migratory effect on MCF-7, irrespective of their concentrations (Figures 2A, B). In contrast to MCF-7, MDA-MB-231 cell migration was significantly reduced only at 10 mM NaB (59.55% ± 6.16%) (Figure 2C), and in a dose-dependent manner at 5 mM (73.68% ± 4.49%), 10 mM (56.4% ± 7.37%), and 15 mM (57.4% ± 5.51%) NaP (Figure 2D). Further investigation into cell invasion showed that NaB and NaP significantly decreased MDA-MB-231 invasion in a dose-dependent manner, with invading cells reaching 46.38% ± 11.01% and 43.83% ± 9.62% at 10 mM NaB and 15 mM NaP, respectively, as compared to the control (Figures 2E, F).

### 3.3 Treatment with NaB or NaP alters the profile of EMT-associated markers in MCF-7 and MDA-MB-231 cells

**FIGURE 3:**
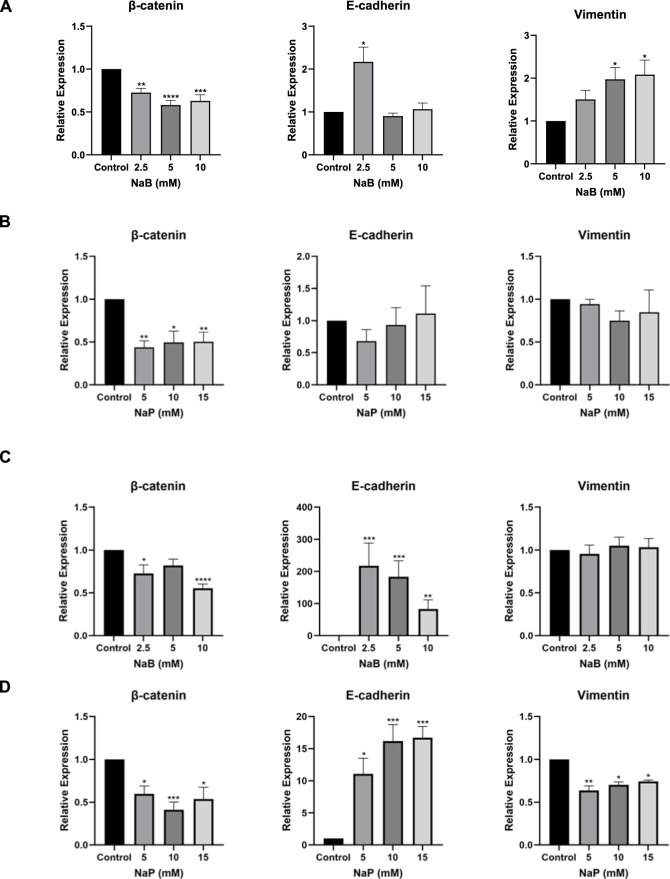
Effect of NaB and NaP on mRNA expression of EMT-associated markers in MCF-7 and MDA-MB-231 using RT-qPCR. Bar graphs showing the relative mRNA expression of β-catenin (left), E-cadherin (middle), and vimentin (right) in MCF-7 **(A, B)** and MDA-MB-231 cells **(C, D)** left untreated or treated with NaB (2.5, 5, 10 mM) or NaP (5, 10, 15 mM) for 24 h. Expression levels were normalized to *GAPDH* mRNA expression. Kruskal–Wallis test followed by Dunn’s multiple comparisons test were used to compare the different concentrations to the control. Values were represented as mean ± SEM from six independent experiments (n = 6), each performed in triplicate. Significant differences were reported as * (p < 0.05), ** (p < 0.01), *** (p < 0.001), or **** (p < 0.0001).

The expression levels of the epithelial marker E-cadherin, mesenchymal marker vimentin, and key EMT regulator β-catenin were evaluated after 24 h of treatment with either NaB (2.5, 5, 10 mM) or NaP (5, 10, 15 mM) in MCF-7 and MDA-MB-231 cells. Treatment with NaB or NaP resulted in a highly significant decrease in β-catenin mRNA expression levels at all concentrations in MCF-7 cells (Figures 3A, B). E-cadherin expression on the other hand was significantly upregulated with 2.5 mM NaB, while no significant increase was observed with NaP. Vimentin mRNA levels were upregulated with the highest NaB concentrations but remained unaffected by NaP. Likewise, both SCFAs significantly downregulated β-catenin mRNA levels in MDA-MB-231 (Figures 3C, D). Interestingly, NaB induced a dramatic increase in E-cadherin levels at all concentrations, suggesting a potential reversal of MDA-MB-231’s mesenchymal phenotype to an epithelial one. NaP also promoted E-cadherin expression, though to a lesser degree than NaB, at 10 and 15 mM. Unlike MCF-7, vimentin mRNA expression was unaffected by NaB but significantly decreased with NaP at all concentrations.

**FIGURE 4:**
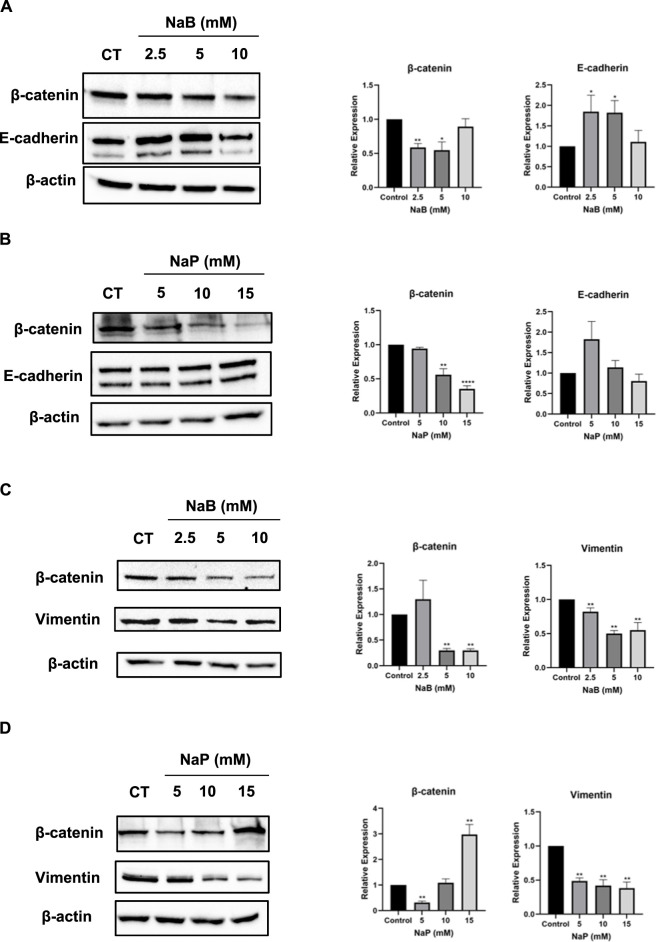
Effect of NaB and NaP on protein expression of EMT-associated markers in MCF-7 and MDA- MB-231 cells using Western blot. Representative blots of β-catenin, E-cadherin, and vimentin in MCF-7 **(A, B)** and MDA-MB-231 **(C, D)** cells left untreated or treated with NaB (2.5, 5, 10 mM) or NaP (5, 10, 15 mM) for 24 h with β-actin as loading control. Adjacent bar graphs display the relative expression levels of these proteins. Kruskal–Wallis test followed by Dunn’s multiple comparisons test were used to compare the different concentrations to the control. Values were represented as mean ± SEM from four independent experiments (n = 4). Significant differences were reported as * (p < 0.05), ** (p < 0.01), *** (p < 0.001), or **** (p < 0.0001).

Similar changes in markers’ expression at the protein level were observed in MCF-7; β-catenin levels were significantly reduced at 2.5 and 5 mM NaB (Figure 4A), and at 10 and 15 mM NaP (Figure 4B). Moreover, E-cadherin levels were significantly increased with 2.5 and 5 mM NaB (Figure 4A), but not with NaP (Figure 4B). Vimentin expression was not detected in MCF-7 cells due to the lack of mesenchymal markers. In MDA-MB-231 cells, β-catenin levels were decreased with 5 and 10 mM NaB or 5 mM NaP (Figures 4C, D). Moreover, NaB and NaP treatments resulted in a significant dose-dependent reduction in vimentin levels (Figures 4C, D). E-cadherin expression was not detected in MDA-MB-231 cells. This can be attributed to the epigenetic suppression of E-cadherin gene (*CDH1*) through promoter methylation in MDA-MB-231 cells (Lombaerts et al., 2006).

**FIGURE 5:**
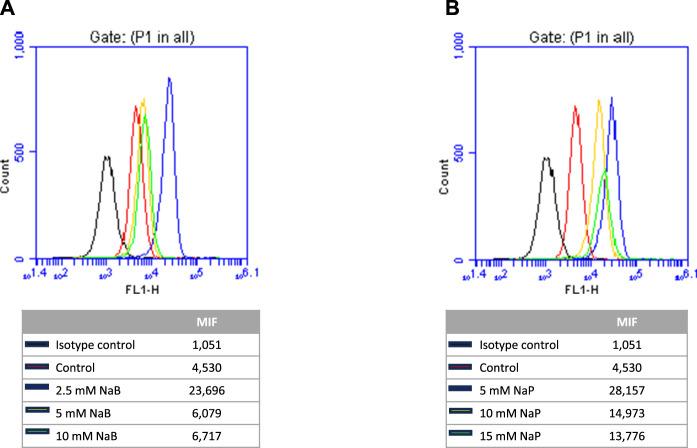
Flow cytometry analysis of E-cadherin expression in MDA-MB-231 cells treated with NaB or NaP. **(A)** Flow cytometry histograms illustrating E-cadherin expression in control (untreated cells, red) and cells treated with 2.5 (blue), 5 (yellow), and 10 mM (green) NaB. **(B)** Flow cytometry histograms illustrating E-cadherin expression in control (untreated cells, red) and cells treated with 5 (blue), 10 (yellow), and 15 mM (green) NaP. Median fluorescence intensity (MFI) was used to quantify E-cadherin expression. Increased MFI values following treatment with either NaB or NaP indicate upregulation of E-cadherin expression in MDA-MB-231 cells.

Since E-cadherin was undetectable in MDA-MB-231 cells via Western blot, we evaluated the effects of NaB and NaP on its expression through flow cytometry analysis. Interestingly, our findings demonstrated an increase in E-cadherin expression in all treatment groups compared to the control. The most substantial increases were observed at the lowest concentrations of NaB (2.5 mM) and NaP (5 mM), with the MFI rising from 4,530 in control cells to 23,696 and 28,157 in the treated cells, respectively (Figures 5A, B). This increase in E-cadherin expression at the protein level is consistent with the previously observed effects at the mRNA level, suggesting that NaB and NaP promote the expression of E-cadherin, which ultimately contributes to the acquisition of epithelial characteristics and a shift towards an epithelial phenotype.

### 3.4 Treatment with NaB or NaP abrogates the nuclear translocation of β-catenin in MCF-7 and MDA-MB-231 cells

**FIGURE 6:**
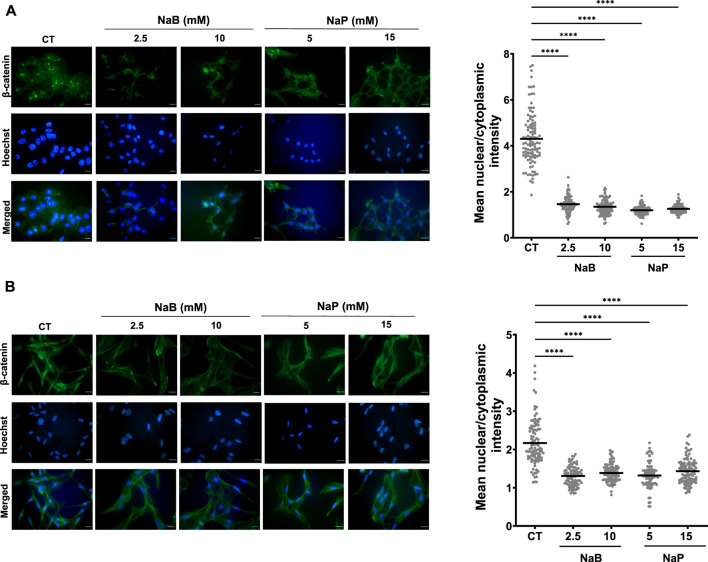
Effect of NaB and NaP on the immunolocalization of β-catenin in MCF-7 and MDA- MB-231 cells. MCF-7 **(A)** and MDA-MB-231 **(B)** cells, either untreated or treated with NaB (2.5 mM and 10 mM) or NaP (5 mM and 15 mM) for 24 h, were fixed and stained with specific antibodies to visualize active (non-phosphorylated) β-catenin in the nucleus. A high level of β-catenin staining is visualized in the nuclei of untreated cells while treated cells exhibit reduced or absent nuclear β-catenin levels. Nuclear and cytoplasmic fluorescent intensities were measured in 10 randomly selected images per experimental condition from three independent experiments (n = 3), yielding data from approximately 100 cells. The mean nuclear-to-cytoplasmic ratios were calculated for each condition, and the data were represented as dot plots where each dot represents an individual cell. One-Way ANOVA test followed by Dunn’s multiple comparisons test were used to compare the different concentrations to the control. Significant differences were reported as * (p < 0.05), ** (p < 0.01), *** (p < 0.001), or **** (p < 0.0001).

Wnt/β-catenin signaling is crucial for cancer progression as it regulates cell proliferation, migration, apoptosis, and other homeostatic aspects (Kim et al., 2019). In breast cancer cells, aberrant Wnt signaling or β-catenin mutations lead to its translocation from the cytoplasm to the nucleus, hence promoting transcription of genes involved in EMT and metastasis, unlike in normal epithelial cells (Wang et al., 2015). In this respect, we aimed to investigate the effects of NaB (2.5 and 10 mM) and NaP (10 and 15 mM) on the nuclear translocation of active (non-phosphorylated) β-catenin in breast cancer cells using immunofluorescence. Interestingly, both compounds were able to abrogate the nuclear translocation of β-catenin, as evident by the reduced amount of fluorescence in the nucleus of treated MCF-7 (Figure 6A) and MDA-MB-231 (Figure 6B) cells compared to the controls.

To further validate these observations, we quantified the mean nuclear-to-cytoplasmic ratio of fluorescence intensities, which confirmed that NaB and NaP treatment significantly reduced the nuclear accumulation of β-catenin in both MCF-7 (Figure 6A) and MDA-MB-231 (Figure 6B) cells, as compared to untreated controls.

### 3.5 Treatment with NaB or NaP modulates ERK and MEK activity in breast cancer cells

**FIGURE 7:**
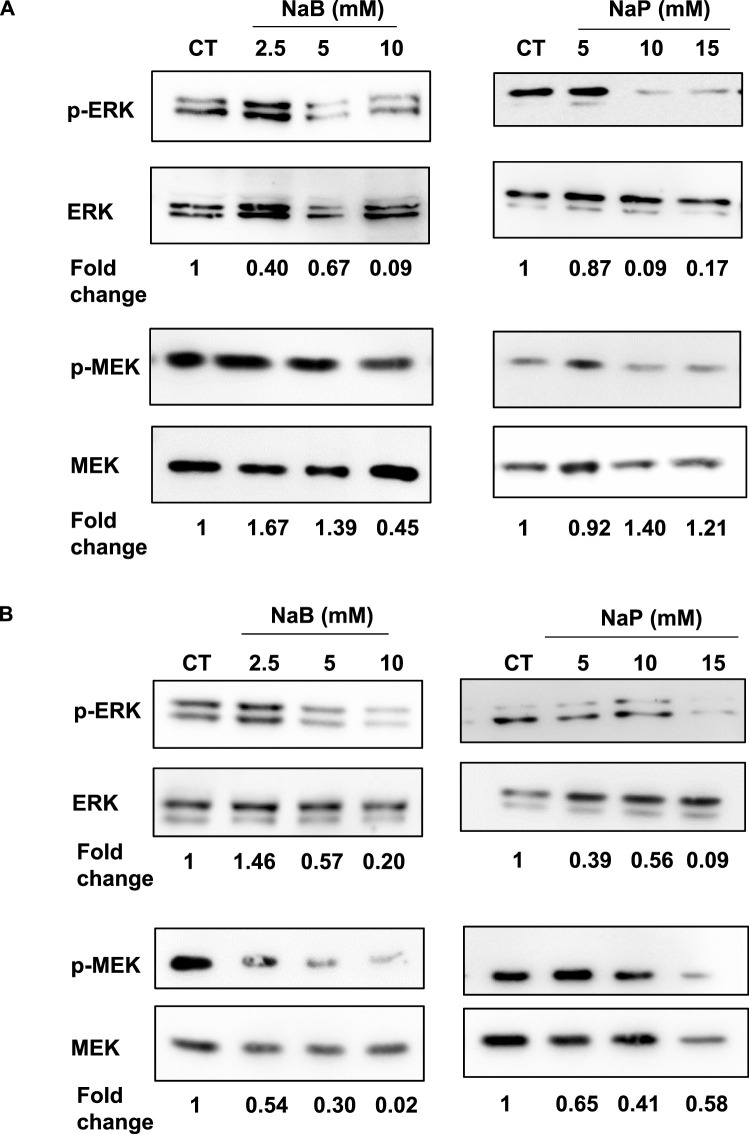
Effect of NaB and NaP on MEK/ERK signaling pathway in MCF-7 and MDA-MB-231 cells. Representative blots showing the expression levels of mitogen-activated protein kinase (MEK), extracellular signal-regulated kinase (ERK), and their phosphorylated (active) forms in MCF-7 **(A)** and MDA-MB-231 **(B)** cells treated with NaB (2.5, 5, 10 mM) or NaP (5, 10, 15 mM) for 24 h. ERK phosphorylation and MEK phosphorylation levels from densitometry were normalized to total ERK and MEK levels respectively, and fold changes relative to the untreated control are indicated below each blot.

The MAPK pathway is the major signal transduction cascade that controls cell growth; its activity is frequently upregulated in different types of cancer, including breast cancer (Sivaraman et al., 1997). Among the three different identified MAPK pathways, the one involving ERK-1 and -2 is the most relevant to breast cancer (Santen et al., 2002). Indeed, previous studies have shown that ERK is critically involved in the progression of TNBC (Borah et al., 2017). Moreover, the activation of the ERK/MAPK signaling pathway was found to promote tumor invasion and metastasis in breast cancer (Li et al., 2023). Finally, it was reported that NaB and NaP are able to inhibit cell invasion via the ERK/MAPK pathway in other cancers, namely, colorectal cancers (Wang et al., 2020). Accordingly, we thought that it is imperative to investigate the ERK/MAPK pathway to better understand the effects of both SCFAs on breast cancer cell invasion and migration. The expression levels of mitogen-activated protein kinase (MEK), extracellular signal-regulated kinase (ERK), and their phosphorylated (active) forms were assessed in MCF-7 and MDA-MB-231 cells by Western blot. Analysis showed that treatment with NaB and, less notably, NaP resulted in decreased levels of phosphorylated ERK and MEK in both MCF-7 (Figure 7A) and MDA-MB-231 cells (Figure 7B). These findings suggest that NaB and NaP mediate their inhibitory effects on breast cancer cell migration by disrupting the ERK1/2 signaling pathway.

## 4 Discussion

Over the years, research has elucidated several key anti-cancer properties conferred by SCFAs. In particular, NaB was found to inhibit cellular proliferation, induce cell cycle arrest, suppress migration and invasion, and trigger apoptosis in various cancers, including colorectal, hepatocellular, and ovarian cancers (Wang et al., 2013; Pant et al., 2017; Xu et al., 2018; Luo et al., 2019; Mrkvicova et al., 2019). However, the role of NaP on these cancer hallmarks remains uncharted territory. Moreover, while some studies have examined the effects of NaB and NaP in human breast cancer, their focus has largely been on cell proliferation, cell cycle regulation, and apoptosis, without addressing the different aspects of metastasis (Chopin et al., 2004; Salimi et al., 2017; Semaan et al., 2020; Park et al., 2021; Ibrahim et al., 2024). This apparent gap prompted us to further investigate the roles played by NaB and NaP in breast carcinogenesis and to elucidate, for the first time, their potential anti-metastatic effects in the context of tumor migration, invasion, and EMT regulation.

Given the heterogeneous nature of mammary carcinomas and the reported differential responses and sensitivity to treatment among the various breast cancer subtypes, our research focused on testing the effects of SCFAs on two phenotypically and molecularly distinct breast cancer cell lines proven to be adequate models for breast cancer investigations: the epithelial-like MCF-7 cell line and the mesenchymal-like MDA-MB-231 cell line. Employing these models in our study allows us to capture a broader spectrum of breast tumor behaviors and any differential responses to treatment with SCFAs.

Interestingly, the previously elucidated cytotoxic effects of NaB and NaP were further validated in 3D spheroid models in the present study. Treatment with either of the SCFAs resulted in a dose-dependent inhibition of spheroid growth, which could be due to inhibition of cell proliferation or a balanced cell proliferation/cell death rate (Garavaglia et al., 2022). The growth impairment by NaB was more pronounced than NaP in both cells as expected. Previous studies have shown that butyrate halts spheroid formation in cholangiocarcinoma (Pant et al., 2017), cervix tumor cells (Dyson et al., 1992), and colorectal cancer (Garavaglia et al., 2022). Additionally, propionate has been shown to synergistically suppress spheroid growth of colorectal cancer with a histone-lysine N-methyl transferase (Ryu et al., 2019). Morphologically, MCF-7 and MDA-MB-231 spheroids became a bit darker in color with treatment, which is indicative of gradual compactness (Hacioğlu and Sakarya, 2018). This increased cohesion between the cells is likely due to the modulation of adhesion markers such as E-cadherin, a key player in facilitating cell-cell adhesion and maintaining tissue integrity (Michels et al., 2009). Thus, an association between E-cadherin and compactness of spheroids can be made, and various studies have supported this notion (Lin et al., 2006; Ivascu and Kubbies, 2007). This is also further supported by our data which signified an upregulation of E-cadherin levels upon NaB treatment in MCF-7 and NaB or NaP treatment in MDA-MB-231 cells.

As for the effect of NaB and NaP on breast cancer cell migration and invasion, our study, to the best of our knowledge, is the first to report that both SCFAs can significantly reduce MDA-MB-231 cell migration and invasion in a dose-dependent manner, suggesting a potential role for these compounds in halting metastatic processes in triple-negative breast cancer subtypes. This observed inhibition of MDA-MB-231 migration contrasts with a lack of a similar effect in the MCF-7 cell line. The absence of such an inhibition in the MCF-7 cell line might be due to the pre-existing low migratory and poorly invasive ability of this model compared to the highly metastatic MDA-MB-231. Nevertheless, a recent study has shown that NaB suppresses both migration and invasion of T47D cells, another luminal A breast cancer cell line (Zhang et al., 2024).

Since EMT is a crucial process in metastasis, we investigated the effects of NaB or NaP on EMT to better understand how these compounds exert their anti-metastatic effects. Our findings revealed a high upregulation of E-cadherin mRNA and protein expression by both NaB and NaP accompanied by a downregulation of vimentin expression by NaB (at protein level only) and NaP (mRNA and protein levels) in MDA-MB-231. These results suggest not only a potential reversal of the EMT phenotype in MDA-MB-231 but also an inhibition of migration upon treatment with NaB and NaP as we had noted earlier. On the other hand, we observed a less pronounced upregulation of E-cadherin mRNA and protein expression levels in NaB-treated MCF-7 cells, while neither expression of E-cadherin nor vimentin was altered in NaP-treated cells. This might explain the absence of inhibition of MCF-7 migration by either compound. It would be of interest to study the expression of vimentin and subsequently migration in mesenchymal-transformed MCF-7 cells in response to treatment, given that MCF-7 cells do not express this marker. In agreement with our findings, previous studies have linked NaB’s inhibition of migration and invasion in different cancer cell lines to EMT regulation. For instance, Wang et al. (2013) showed that NaB inhibits migration and invasion in hepatocellular carcinoma cells by blocking HDAC4 and TGFβ1-induced EMT. This aligns with findings in ovarian cancer, where HDAC4 silencing reduced cell migration (Ahn et al., 2012), and in colorectal cancer, where NaB inhibited EMT via miR-200, increasing E-cadherin and reducing Snail and vimentin expressions (Xu et al., 2018). Wang et al. (2020) further reported that NaB impairs migration in colorectal cancer by targeting Thioredoxin-1, an EMT inducer, leading to decreased vimentin and N-cadherin and increased E-cadherin expressions. Interestingly, Thirunavukkarasan et al. (2017) revealed that SCFAs, by interacting with their FFA receptors, drive MDA-MB-231 cells toward a non-invasive phenotype by upregulating E-cadherin expression. Similar to our study, these effects were absent in MCF-7 cells.

Furthermore, treatment with NaB or NaP downregulated β-catenin and abrogated its translocation from the nucleus to the cytoplasm in both cells, suggesting a potential inhibitory effect of the Wnt/β-catenin pathway. This observation aligns with Vincan et al. (2000), which showed that NaB treatment resulted in stabilization of membrane-bound β-catenin in colon cancer cells, promoting their differentiation and cell-cell adhesion.

Finally, our findings showed that NaB, and less extensively NaP, reduced ERK and MEK phosphorylation in MCF-7 and MDA-MB-231 cells. The ERK signal transduction cascade is aberrantly overactivated in several tumors, including breast cancer (Santen et al., 2002). Activation of MAPK is a factor for regulating cell migration as ERK1/2 controls the motility-dependent activation of myosin light chain kinase, FAK, and calpain (Sugiura et al., 2021). Previous reports have demonstrated the ability of butyrate and propionate to inhibit colon cancer cellular proliferation (Zeng et al., 2017) or motility (Li et al., 2017) *via* decreasing ERK1/2 phosphorylation, which aligns with our observations.

In conclusion, our findings notably reveal the anti-migratory, anti-invasive, and EMT regulatory potential of NaB and NaP in breast cancer cells. More importantly, we also identified the Wnt/β-catenin and ERK/MEK signaling pathways as potential molecular mechanisms by which these SCFAs exert their effects, thus rendering them potential players in the development of targeted therapeutic strategies against breast cancer. Nevertheless, future research should consider extending our findings to *ex vivo* and *in vivo* studies to fully capture the effects of NaB or NaP and understand their translational potential. Moreover, it would be intriguing to study *CDH1* promoter hypermethylation as part of the EMT program, which may be responsible for the more aggressive phenotype of breast cancer, thus enabling metastasis. Furthermore, the precise mechanisms and the crosstalk between the various signaling pathways are remaining facets that beckon further exploration.

## Data Availability

The original contributions presented in the study are included in the article/supplementary material, further inquiries can be directed to the corresponding author.
